# Tailoring EHRs for Specific Working Environments Improves Work Well-Being of Physicians

**DOI:** 10.3390/ijerph17134715

**Published:** 2020-06-30

**Authors:** Suvi Vainiomäki, Tarja Heponiemi, Jukka Vänskä, Hannele Hyppönen

**Affiliations:** 1 Department of Clinical Medicine, University of Turku, 20014 Turku, Finland; 2 Turku Welfare Division, 20100 Turku, Finland; 3 Department of Health and Social Care Systems, Finnish Institute for Health and Welfare, 00271 Helsinki, Finland; tarja.heponiemi@thl.fi (T.H.); hannele.hypponen@thl.fi (H.H.); 4 Finnish Medical Association, 00271 Helsinki, Finland; jukka.vanska@laakariliitto.fi

**Keywords:** electronic health records, work–life balance, workflow

## Abstract

Electronic health records (EHRs) have an impact on physicians’ well-being and stress levels. We studied physicians’ experiences with EHRs and their experienced time pressure and self-rated stress by an electronic questionnaire sent to Finnish physicians aged under 65 in 2017. Our sample was 2980 physicians working in the public sector, health care centers (35.5%) or hospitals (64.5%). Experienced technical problems were positively associated with experienced time pressure, whereas user-friendliness of the EHRs was negatively associated with experienced time pressure. Low perceived support for internal cooperation was associated with high levels of time pressure in hospitals. Those experiencing high levels of technical problems were 1.3 times more likely to experience stress compared to those experiencing low levels of technical problems. Better user-friendliness of the EHRs was associated with lower levels of self-rated stress. In both working environments but more strongly in primary health care, technical problems were associated with self-rated stress. Technical problems and user-friendliness of EHRs are the main factors associated with time pressure and self-rated stress. Health care environments differ in the nature of workflow having different demands on the EHRs. Developing EHR systems should consider the special needs of different environments and workflows, enabling better work well-being amongst physicians.

## 1. Introduction

Electronic health records (EHRs) present a threat to physicians’ work well-being. Finnish physicians graded their EHRs below average, and there have not been significant improvements in the average grades during years 2010, 2014, 2017 [1,2,3,4,5,6]. A longitudinal 9-year study found the strain still increasing, especially in primary care environment and among those in leading positions [7]. However, in hospital environments, there was a plateau from 2010 to 2015. A study from the same follow-up data found that cognitive workload predicted stress related to poorly functioning information systems (ISs), as did poor teamwork and time pressure. Job satisfaction on the other hand predicted less stress [8].

Our previous study from a survey in 2014 with a similar setting examined the factors influencing time pressure and job control. Technical properties and usability emerged as the main factors, with more detailed factors being fewer login procedures, easier readability of nursing records and decreased need for separate documentation for statistical purposes. Physician participation in the EHR development was associated with the higher feeling of job control and lower time pressure [9].

Physician well-being and work satisfaction have been studied widely [10,11,12]. Mental stress and burnout emerge in physicians more often than in the general population: for example, one fifth of Finnish physicians considered burnout as a present threat in 2015 [8,13]. Finnish primary care physicians are more strained than their peers as work is more solitary and the pace is often extensive. This has led to an increase in experienced feelings of hurry and patient-related stress especially in Finnish primary care physicians during the last decades [14,15,16]. Private sector physicians are more satisfied with their work environments and their EHRs [17,18,19,20].

Work well-being is influenced by many extrinsic but also intrinsic factors [21]. Organizational interventions present key means to promote work well-being, but comprehensive leadership can also influence the intrinsic factors. Tawfik et al. [22] pointed out four organizational opportunities to enhance physician well-being: “developing leaders, cultivating community and organizational culture, improving practice efficiency, and optimizing administrative policies.” They claim that reducing the burden caused by documentation and improving practice workflows and efficiency balance the demands and resources of the employee. Haque et al. proposed that work well-being can be influenced by responsible leadership and organizational commitment, thus gaining boarder benefits with respect to employees, organizations and stake holders [23,24].

Colin et al. pointed out that the right use of electronic health record advances has the potential to improve overall health but, when adversely affecting physician well-being, poses a threat for the efficacy and autonomy of a physician and, hence, to patient care and safety: “The Quadruple Aim would ensure that changes to the healthcare system optimally serve the entire system, including individual patients, populations, and the professionals engaged in delivering care.” [25] 

EHRs have been found as a stress factor in physician work in many countries and specialties. An American study on physicians found that dissatisfaction with the EHR was associated with intent to reduce clinical work hours and to leave the current practice [26]. A Canadian study on family physicians’ job satisfaction found that the use of an EHR was negatively associated with both processional and work–life balance satisfaction [27], and an American study found that an increase in satisfaction on EHR use increased also job satisfaction among family physicians [28]. An American study found electronic medical record requirements as one of the six main themes emerging amongst qualitative interviews of 26 family physicians about job satisfaction and burnout [29].

Another American study found that primary care physicians using a moderate number of functions reported more stress and less job satisfaction than physicians with low numbers of EHR functions [30]. The researchers suggested that this was because of the transition phase of EHR adoption. A study found that neurologists experienced their EHRs taking time away from the patient and actual care and mainly being used for administrational purposes [31]. A study on physician residents found a strong positive correlation between EHR use and resident burnout [32]. It seems clear that EHRs have a negative influence on physician job satisfaction and burnout, but it is not clear how we could change this for the better.

Physicians’ work includes complex and demanding activities such as multitasking, clinical reasoning, problem-solving, and a need to deal with vast amounts of information. The Information Chaos Theory conceptualises information overload, underload, scatter, conflict and erroneous information as information chaos [33]. High load from poorly functioning EHRs may result in a situation where professionals have fewer resources and capacities to cope with them and experience more stress and lack of time for their daily work. Thus, poorly functioning EHRs may predispose physicians to time pressure and stress, and this may vary according to working environment, for example, due to differences in information chaos in the work environment. In light of these theoretical frameworks, we examined the associations of EHR-related variables with time pressure and stress and how these associations differed according to working environment.

## 2. Materials and Methods

### 2.1. Context

In Finland, public health care uses EHRs [34]. Finnish health care is mainly public, is tax-payed and is supplemented by the private sector. The majority of Finnish physicians (70%) work in the public sector, and the rest work in the private sector [35]. Primary care is given in municipal health centers, whereas secondary and tertiary care are given in hospitals. Two thirds of public sector physicians work in hospitals, and the rest work in health centers [35]. In hospitals, multi-professional cooperation is continuous, whereas in health centers, physicians work in more solitude settings, though there are also nurses, physiotherapists, and other health care personnel working in the same facilities. In primary health care, the appointments are mainly short visits, whereas in hospitals, the patient can be referred many times during a period of treatment.

Physicians’ experiences with EHRs were surveyed by an electronic questionnaire in 2017, the target population being Finnish physicians aged less than 65 and working in clinical work (*n* = 19.627). We studied only the more strained public sector physicians in hospital and health center environments and excluded private physicians. The questionnaire is available in English [36]. The questionnaire is a part of an ongoing survey (2010, 2014 and 2017) which focuses on physician work and experiences with EHR functioning [1,2,3,5,6,9,37]. The questions focused on usability of the systems.

As a behavioral study with compiled information on the social background and work history placed on the market and physicians’ experiences on EHR usability and work well-being, we did not collect sensitive, potentially harmful information about the participants. The autonomy of research subjects was respected. There was informed consent, and no harm was possible for the subjects. Confidentiality of the subjects and research data are protected. According to Finnish legislation, no ethical assessment or approval is mandatory for a study such as this. The Finnish law (Medical Research Acts 1999/488, 2004/295, and 2010/794) states medical research requiring the approval of an appropriate ethics committee as follows: research involving intervention in the integrity of a person, human embryo or human fetus for the purpose of increasing knowledge or the nature of diseases in general. Also, according to the local and national ethical instructions for research this study did not require ethical approval [38].

Time pressure was measured with two items measuring how often (during the past half-year period) a person had been distracted by, worried about or stressed about (1) being in a constant hurry and time pressure coming from unfinished work tasks and (2) having too little time to do work properly. The items were rated on a five-point Likert scale ranging from 1 (never) to 5 (very often), and higher scores indicated higher time pressure. Self-rated stress was measured with a widely used single-item self-rated stress measure [39]: “Stress means a situation when a person feels tense, restless, nervous or anxious or is unable to sleep at night because his or her mind is troubled all the time. Do you feel that kind of stress these days?” Response options were not at all/just a little/to some extent/quite a lot/very much. For the analyses, these were categorized as 0 = not at all/just a little and 1 = to some extent/quite a lot/very much.

We used the same grouping of seven factors as in our previous study, done by factor analysis of the 36 usability questions [9]. The factor groups are the following with the number of usability questions included in each in brackets: user-friendliness (9), benefits (7), technical problems (6), feedback (4), and internal cooperation (3). External cooperation (5) and results (2) were not included in the analyses. Other variables measured were age, specialist status, employment sector, number of systems in daily use and experience in using current EHR.

### 2.2. Statistical Analyses

The associations of independent variables with time pressure were analyzed with analyses of covariance. The analyses of covariance were chosen because the dependent variable was a continuous variable and independent variables included both continuous and categorical variables; thus, this method was suited well for analyses regarding time pressure. The analysis model included age, gender, working environment, specialization status, number of systems in daily use, experience in using current EHR, technical problems, perceived benefits, feedback, internal cooperation and user-friendliness as independent variables. Because user-friendliness and technical problems correlated highly with each other (*r* = −0.63), user-friendliness was examined in a separate analysis to avoid multicollinearity (with the same model without technical problems). The associations of independent variables with binary self-rated stress were examined with logistic regression analyses with the same models as with the analyses of covariance mentioned above. This analysis method was chosen because the dependent variable was binary variable and logistic regression is a suitable method for analysing binary outcomes.

The interactions of working environment with IS-related variables (number of systems in daily use, experience in using current EHR, technical problems, perceived benefits, feedback, internal cooperation and user-friendliness; in separate analyses) were examined with analyses of covariance for time pressure and logistic regression for self-rated stress adjusted for age, gender and main effects.

## 3. Results

### 3.1. Demographics

Altogether, 4018 physicians answered the survey. Two thirds were women, and two thirds were specialists. In comparison to eligible Finnish physician population, women and older physicians are a little overrepresented in the sample. However, the results can be generalized to the Finnish physician population [35]. The present sample includes physicians working in the public sector, either in hospitals (*n* = 1943, 64.5%) or in health centers (*n* = 1070, 35.5%). Seventy-five percent regarded themselves as experienced users. Almost half of the physicians self-rated their stress levels to be at least at the moderate level. (Table 1). The used questionnaire has been tested for validity, and the comparison of the respondents with the target population represents the sample in 2017 as described in a previous article [37].

### 3.2. Time Pressure

The analyses of covariance (Table 2) showed significant associations of working environment, gender, technical properties and user-friendliness with time pressure. Technical problems had the strongest association with time pressure, but user-friendliness also showed strong association. Experience of time pressure was higher in health centers and among women. We did not find significant associations regarding the age of the physician, specialty, number of clinical systems in daily use, perceived benefits, experience of the physician as an EHR user or how feedback to the system supplier works.

### 3.3. Self-Rated Stress

The logistic regression analysis (Table 3) showed that female gender and technical problems were associated both with almost 1.3 times higher likelihood of self-rated stress. Internal cooperation was associated with 0.9 times lower likelihood, and user-friendliness was associated with 0.8 times lower likelihood of self-rated stress.

### 3.4. Interactions of Working Environment with IS-Related Variables

The interaction between working environment and internal cooperation was significant for time pressure (F = 4.55, *p* = 0.033). In hospitals, low internal cooperation was associated with high time pressure (F = 25.39, *p* < 0.001), whereas in primary health care, the association was not significant (F = 1.78, *p* = 0.182). The interaction between working environment and technical problems was significant for self-rated stress (Wald’s χ = 11.84, *p* = 0.001). Technical problems were related to self-rated stress in both working environments but more strongly in primary health care (OR = 1.50, 95% CI = 1.32–1.71) than in hospitals (OR = 1.14, 95% CI = 1.04–1.25). Other interactions between working environments and IS-related variables were not significant.

## 4. Discussion

Technical problems and user-friendliness of the EHRs are associated with physicians’ time pressure and stress. This supports our previous findings that EHR factors are related to the work well-being of physicians [8,9]. With stabile and user-friendly ISs, we could ease the time pressure and stress that physicians are experiencing. It has been previously found that time pressure and stress are more likely experienced in health center environments [14,15,16], but our study indicates that technical problems of the EHRs is one of the factors contributing to this stress.

Technical problems cause delays, interruptions and more work for physicians and, according to our findings, result in time pressure and stress. Our findings are congruent with a previous finding showing that technical characteristics of the IS, such as the reliability, response time and functionality, emerged as the most important factors associated with user satisfaction [40]. In a primary care setting, self-rated stress is more strongly associated with technical problems than in a hospital setting. In previous studies, there has not been a clear difference between hospital and primary care environments concerning technical instabilities or slow reactions to input [3,5,6]. The association between self-rated stress and technical problems in our study was significant in both environments but more so in primary care setting. The structure and organization of work in health centers and hospitals produces a different workflow for the physician, and daily work is very different from each other. In health centers, there is a certain pace given by the appointment book and patients are seen only for a while. In hospitals, the patients tend to be sicker and more demanding to care for, but there is a certain elasticity to the workflow as the patients are usually present for longer periods. An EHR acts as a supporting or distracting factor for the workflow, and this influences work well-being. However, perhaps being overall more stressed [13,14,17,18], primary health care physicians also experience technical problems more as a strain.

According to our findings, it seems that user-friendliness of the EHRs results in less time pressure. User-friendliness consists of usability such as logical functions, understandable terminology, straightforward routine tasks, and easy and smooth entering and use of patient information [1]. EHRs should guide and help users throughout the tasks performed and not require massive training. Possible mistakes should be easily corrected. When use of a software is easy, physicians’ workflow is better and more time may be found for direct interaction with the patient and for treatment procedures.

In a previous article [9], we found that association between user-friendliness problems and time pressure varied according to working environment, such as understanding nursing documents in hospitals and documentation of patient information for statistical purposes in health centers. Again, different workflows in different working environments demand software designs that support work in their corresponding environments. All in all, primary care use of EHRs differs from hospital use and more insight is needed on workflow and EHR use of primary care physician in order to relieve the strain they are experiencing.

We found that internal cooperation was more strongly associated with time pressure in hospitals than in health centers. The requirements for internal cooperation for an EHR in a hospital setting are more demanding: hospital physicians need more cooperation, as physicians work closely with other physicians, nurses and other health care professionals, whereas in health centers, there is less interaction. A study by Unni et al. researched and identified high-level cognitive tasks that were associated with end-user satisfaction and identified that easy and efficient communication and coordination between various clinical teams is associated with significantly higher user satisfaction amongst clinicians [41]. They suggest that the ability of an EHR to support particularly these high-level cognitive tasks could lead to better user satisfaction. Quinn et al. not only found EHR data fragmentation and one-way communication to be barriers of diagnostic reasoning but also saw EHRs as an opportunity to enhance information sharing and diagnostic reasoning with software platform and interface improvements [42].

It is notable that different health care environments have different demands on EHRs, and thus different workflow can either be supported or distracted by EHRs. In order to support the complex cognitive workflows of health care environments, supporting technology must be designed to adapt the demands of the work and the cognitive strategies employed by health care practitioners [43]. Primary health care needs for an EHR are very different from hospital needs [44]. By developing systems that work well in the intended working environment, one could gain benefits, such as better physician well-being and others including efficient procedures and workflow. User-friendliness diminishes the likelihood of time pressure and stress. Ease of using the system helps physicians to operate their EHRs as part of the consultation and not as the aim of the consultation. Zheng et al. claim that studying workflows and workarounds during and after IS implementation can improve health system performance and patient safety and can offer recommendations on how to conduct such studies [45].

Perceived benefits, feedback from EHR vendors and experience in using the system did not significantly affect time pressure or stress in our study. This is partly contrary to previous findings showing the importance of experience in using EHRs for the well-being of physicians [7].

The survey was cross-sectional, so it was not possible to study change in between years. Other longitudinal studies have proven that there is an increase in physicians’ her-related stress [8] so we concentrated on the factors influencing stress. Because we used self-reported measures, problems associated with an inflation of the strengths of relationships and with common method variance are possible. In addition, although we controlled for many factors, the possibility of residual confounding cannot be totally ruled out. Moreover, the total number of respondents in the total survey was rather large, being about 4000, but the response rate remained relatively low (22%); thus, generalizability of the findings to all physicians should be done with caution. However, a comparison with the target population showed good representativeness of the sample [6,35,37].

We had only one question measuring overall stress and only two items to measure work-related time pressure. More questions on these variables would have been more efficient in differentiating these variables, which can easily be mixed with each other. However, these measures have previously been used widely and single-item stress measure has been a proven valid and reliable measure of stress [39]. The overall questionnaire offered no possibilities to concentrate on work well-being more closely.

## 5. Conclusions

EHR systems influence physician work well-being, and this should not be overlooked. As EHR systems are constantly developing, developers and implementing organizations should concentrate on the factors that influence work well-being and stress. Stability, ease of overcoming technical problems and user-friendliness should be of main concern. Stability of the software does not depend solely on the vendor: the implementing health care organizations are responsible for the stability of the hardware, the load of networks and the enterprise architecture. The usability of the software is connected to physicians’ workflow, and EHR designs should support effective and efficient care of patients in their specific user environments.

## Figures and Tables

**Table 1:** Characteristics of the study sample.

Characteristics	*n*	%
Gender		
Women	1975	66.3
Men	1005	33.7
Employment Sector		
Hospital	1943	64.5
Primary Health Care	1070	35.5
Specialist Status		
No	1052	34.9
Yes	1961	65.1
Systems in Daily Use		
1–2	1616	54.5
3 or More	1351	45.5
Experience in Using Current EHR		
Beginner	806	26.9
Experienced	2193	73.1
Self-Rated Stress		
Low	1598	53.6
High	1384	46.4
	Mean	SD
Age	45.08	11.12
Time Pressure ^a^	3.77	0.97
Technical Problems ^a^	2.93	0.83
Perceived Benefits ^a^	2.77	0.78
Feedback ^a^	2.19	0.88
Internal Cooperation ^a^	3.43	0.86
User-Friendliness ^a^	2.76	0.78

^a^ The scale ranged between 1 and 5.

**Table 2:** The results of the analyses of covariance for time pressure.

Variables	F	*p*-Value
Gender	24.73	<0.001
Age	0.22	0.638
Employment Sector	49.39	<0.001
Specialization ^a^	0.55	0.458
Number of Systems in Daily Use ^b^	0.00	0.993
Experience in Using Current HER ^c^	0.01	0.923
Technical Problems	31.67	<0.001
Perceived Benefits	1.13	0.288
Feedback	0.00	0.989
Internal Cooperation	2.05	0.152
User-Friendliness	16.00	<0.001

^a^ Coded as 0 = not specialized or specialization ongoing and 1 = specialist; ^b^ Coded as 0 = 1–2 systems and 1 = 3 or more systems; ^c^ Coded as 0 = beginner and 1 = experienced.

**Table 3:** The results of the logistic regression analysis for self-rated stress, Odds Ratios (ORs) and their 95 percent Confidence Intervals (95% CIs).

Variables	OR	(95% CI)	*p*-Value
Gender			0.002
Men	1		
Women	1.29	1.10–1.52	
Age	1.00	0.99–1.00	0.273
Employment Sector			0.350
Hospital	1		
Primary Health Care	1.08	0.92–1.28	
Specialist Status			0.848
No	1		
Yes	1.02	0.83–1.25	
Number of Systems in Daily Use			0.943
1–2	1		
3 or More	1.01	0.86–1.18	
Experience in Using Current EHR			0.143
Beginner	1		
Experienced	0.88	0.74–1.04	
